# Peripapillary Retinoschisis in Glaucomatous Eyes

**DOI:** 10.1371/journal.pone.0090129

**Published:** 2014-02-28

**Authors:** Eun Ji Lee, Tae-Woo Kim, Mijin Kim, Yun Jeong Choi

**Affiliations:** Department of Ophthalmology, Seoul National University College of Medicine, Seoul National University Bundang Hospital, Seongnam, Korea; Medical Faculty Mannheim of the University of Heidelberg, Germany

## Abstract

**Purpose:**

To investigate the structural and clinical characteristics of peripapillary retinoschisis observed in glaucomatous eyes using spectral-domain optical coherence tomography (SD-OCT).

**Methods:**

Circumpapillary retinal nerve fiber layer (cpRNFL) and macular cross-hair SD-OCT scans and infrared fundus images of the glaucoma patients from the Investigating Glaucoma Progression Study (IGPS) and healthy volunteers were reviewed. Optic disc images obtained using enhanced depth imaging (EDI) SD-OCT were also evaluated. The structural characteristics and clinical course of the retinoschisis associated with glaucoma were investigated.

**Results:**

Twenty-five retinoschisis areas were found in 22 of the 372 patients (5.9%) included in the IGPS, and in 1 area in 1 of 187 healthy control subjects (0.5%). In the 22 glaucomatous eyes with retinoschisis, the schisis was attached to the optic disc and overlapped with the retinal nerve fiber layer (RNFL) defect. The RNFL was the layer most commonly affected by the retinoschisis, either alone or together with other deeper layers. Acquired optic disc pit was identified in 8 eyes on disc photography and/or B-scan images obtained by EDI SD-OCT. Spontaneous resolution of this condition was observed in nine eyes. No retinal detachment or macular involvement of the retinoschisis was observed in any of the eyes. Multivariate analysis showed a significant influence of a higher intraocular pressure at SD-OCT scanning on the presence of retinoschisis (Odds ratio  = 1.418, P = 0.001).

**Conclusions:**

The present study investigated 22 cases of peripapillary retinoschisis in glaucomatous eyes. The retinoschisis was attached to the optic nerve and topographically correlated with RNFL defect. It often resolved spontaneously without causing severe visual disturbance. Care should be taken not to overestimate the RNFL thickness in eyes with retinoschisis, and also not to misinterpret the resolution of retinoschisis as a rapid glaucomatous RNFL deterioration.

## Introduction

Retinoschisis of the macula and the peripapillary area has been described in various entities including X-linked retinoschisis, congenital optic disc abnormalities such as optic disc pit and optic disc coloboma, and myopia. X-linked retinoschisis can be progressive and is often associated with significant visual loss.[1] Furthermore, it often accompanies retinal detachment and/or vitreous hemorrhage, which generally necessitates surgical interventions to prevent or treat the associated complications.[2], [3] Retinoschisis associated with a congenital optic disc abnormality is also often complicated with retinal detachment, which requires laser photocoagulation[4] or pars plana vitrectomy and gas tamponade.[5] Retinoschisis associated with myopia is generally stable but can be accompanied by foveal detachment, which may necessitate surgical intervention.[6]

Several recent studies have documented the observation of retinoschisis in glaucoma patients, the clinical features of which were variable, ranging from spontaneous resolution[7], [8] to retinal detachment that required surgical intervention.[9] However, the reported features were based on either a single case report or small series involving up to only five patients. Thus, the clinical characteristics and importance of the retinoschisis associated with glaucoma remain to be elucidated. In addition, each report has described retinoschisis in patients with different types of glaucoma, including primary open-angle glaucoma (OAG), normal-tension glaucoma, intermittent angle closure glaucoma, and narrow-angle glaucoma, and this has resulted in the reports suggesting different pathogenic mechanisms. Further studies are therefore needed to clarify the underlying mechanism.

The purpose of the present study was to determine the structural and clinical characteristics of peripapillary retinoschisis associated with glaucoma in a relatively large sample.

## Methods

### Ethics statement

The investigation was based on a review of the images of patients included in the Investigating Glaucoma Progression Study (IGPS), which is an ongoing prospective study at the Glaucoma Clinic of Seoul National University Bundang Hospital (SNUBH). Healthy subjects were also included as a control. The study was approved by the Institutional Review Board of SNUBH and conformed to the Declaration of Helsinki. Written informed consent to participate in the study was obtained from all patients.

### Study subjects

The database of patients included in the IGPS between September 2009 and July 2013 was reviewed. The aim of the IGPS is to measure the rate of progression in OAG and to determine the factors associated with a fast progression of OAG. OAG was defined as the presence of glaucomatous optic nerve damage (neuroretinal rim notching or thinning, or retinal nerve fiber layer [RNFL] defect) and associated visual field defects without ocular disease or conditions that may elevate the intraocular pressure (IOP). A glaucomatous visual field defect was defined as (1) outside normal limits on the glaucoma hemifield test or (2) three abnormal points with *P*<5% of being normal, one with *P*<1% by pattern deviation; or (3) a pattern standard deviation of <5% if the visual field was otherwise normal, as confirmed on two consecutive tests.

Patients enrolled in the IGPS underwent a complete ophthalmic examination including visual acuity assessment, refraction, slit-lamp biomicroscopy, gonioscopy, Goldmann applanation tonometry, and dilated stereoscopic examination of the optic disc. They also underwent central corneal thickness measurement (Orbscan II, Bausch & Lomb Surgical, Rochester, NY, USA), axial length measurement (IOL Master version 5, Carl-Zeiss Meditec, Dublin, CA, USA), stereo disc photography, red-free fundus photography, infrared (IR) fundus photography, enhanced depth imaging (EDI) spectral-domain optical coherence tomography (SD-OCT; Spectralis OCT, Heidelberg Engineering, Heidelberg, Germany) scanning of the optic disc, macular cross-hair scan, and circumpapillary retinal nerve fiber layer (cpRNFL), and standard automated perimetry (Humphrey Field Analyzer II 750; 24-2 Swedish interactive threshold algorithm; Carl-Zeiss Meditec).

All patients included in the IGPS had been followed up with IOP and SD-OCT RNFL thickness measurement every 4–6 months, and with optic disc scanning using EDI SD-OCT at intervals of 1–2 years.

Healthy controls were enrolled by advertisement and by invitation from patients with incipient cataract or dry eye. The inclusion criteria for the control group were eyes with an IOP below 22 mmHg without anti-glaucoma medication, normal-appearing optic discs, and normal visual fields. A normal-appearing optic disc was defined as the absence of glaucomatous optic neuropathy and pallor or swelling of the optic disc. A normal visual field was defined as the absence of glaucomatous visual-field defects and neurologic field defects. Healthy control patients were matched with glaucoma group in terms of age and axial length.

For both glaucoma patients and healthy controls, eyes with a visual acuity of <20/40, a spherical refraction of <−12.0 or >+3.0 diopters, and a cylinder correction of >±3.0 diopters were excluded. Eyes having optic disc anomalies including congenital optic disc pit or coloboma, intrachoroidal cavitation,[10] a history of ocular surgery other than cataract extraction and glaucoma surgery, or intraocular disease (e.g., uveitis, central serous chorioretinopathy, retinal vasculitis, diabetic retinopathy or retinal vein occlusion) or neurologic disease (e.g., pituitary tumor) that could cause visual field loss were also excluded.

### Spectral-domain optical coherence tomography

The SD-OCT examination included IR imaging of the optic disc and peripapillary area, cpRNFL scan, macular cross-hair scan, and volumetric optic disc scans performed using the EDI technique. The details of the protocol for scanning of the optic nerve using EDI SD-OCT to evaluate the lamina cribrosa (LC) are described elsewhere.[11]–[13] In brief, approximately 75 horizontal B-scan section images covering the optic disc, 30–34 µm apart (the scan-line distance being determined automatically by the instrument), were obtained for each eye. For each section, 42 OCT frames were averaged, which provided the best trade-off between image quality and patient cooperation.[12]

### Defining the retinoschisis and its circular extent and depth of retinoschisis

**Figure 1:**
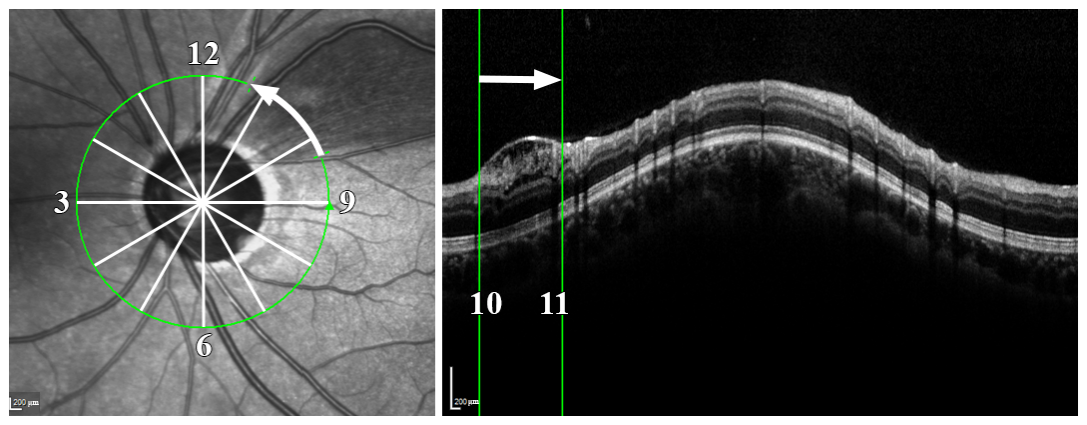
Measurement of the clock-hour location and extent of the retinoschisis. The superior clock hour was 12 o'clock; the others were assigned in a clockwise manner in the right eye and counterclockwise in the left.

Peripapillary retinoschisis was defined when the splitting of the inner or outer retinal layers adjoined the optic disc margin in the cpRNFL SD-OCT B-scans. The circular extent of the retinoschisis was determined based on IR and cpRNFL B-scan images. The clock-hour extent of the retinoschisis area at the OCT scan circle was determined using the Heidelberg Eye Explorer (version 1.7.1, Heidelberg Engineering), which allowed navigation of the corresponding locations between the cpRNFL B-scan images and the IR fundus images. The superior clock hour was 12 o'clock; the others were assigned in a clockwise manner in the right eye and counterclockwise in the left (Figure 1).

### Statistical analysis

Clinical characteristics between groups were compared using independent samples t-test or chi-square test. Factors influencing the retinoschisis were assessed using logistic regression analysis. Statistical analyses were performed using PASW Statistics 18.0.0 software (SPSS, Chicago, IL, USA). A P value of less than 0.05 was considered statistically significant.

## Results

The OCT images of 372 glaucoma patients who were included in the IGPS and 187 healthy control subjects were reviewed. There was no difference in age, gender, axial length and central corneal thickness between the glaucoma and control group (Table 1).

**Table 1:** Clinical characteristics of glaucoma group and healthy control group.

	Glaucoma group (n = 372)	Control group (n = 187)	P-value*
Age (years)	55.0±13.3	54.5±15.9	0.676
Gender (M/F)	185/187	91/96	0.812
IOP at SD-OCT scanning (mmHg)	12.2±3.3	12.8±3.1	0.087
Visual field MD (dB)	−5.6±6.8	−0.2±1.5	**<0.001**
Axial length (mm)	24.4±1.3	24.2±1.1	0.159
Central corneal thickness (µm)	554.1±47.7	558.2±38.5	0.389

* Comparisons were performed using independent samples t-test for continuous variables and chi-squire test for categorical variables.

Values are shown in mean ± SD.

Statistically significant values are shown in bold.

IOP - intraocular pressure, MD - mean deviation, PSD - pattern standard deviation.

In glaucoma group, 22 patients (5.9%) exhibited peripapillary retinoschisis on cpRNFL SD-OCT B-scan images. In the control group, retinoschisis was significantly less frequent (0.5%, 1/187) (P = 0.003, chi-square test). Of the 22 OAG eyes with retinoschisis, 1 had 2 schisis areas in the superonasal and inferotemporal sectors, and 1 had 3 schisis areas in the superonasal, inferonasal and inferotemporal sectors. Thus, a total of 25 localized retinoschisis was found in the 22 eyes. Individual clinical characteristics of the 22 OAG patients with retinoschisis are given in Table 2.

**Table 2:** Clinical characteristic of 22 glaucoma patients with retinoschisis.

Case No	BCVA	uIOP	MD (dB)	F/U duration (m)*	APON on stereo disc photography	Pit on SD-OCT B-scan	Resolution
1	20/20	12	−0.14	36.2	+	+	-
2	20/20	16.5	−6.37	27.4	+	+	+
3	20/20	18	−10.29	31.0	+	+	+
4	20/25	27	−22.59	34.3	-	-	-
5	20/15	27.5	−12.53	34.3	-	-	-
6	20/30	30	−30.41	7.9	-	-	-
7	20/25	16	−4.04	38.5	-	-	-
8	20/30	15	−7.53	14.2	-	-	+
9	20/20	15.4	−3.70	34.7	-	-	+
10	20/20	15	−3.35	34.5	+	+	+
11	20/30	14.3	−7.10	0	+	-	-
12	20/20	24.5	−22.78	3.5	-	-	+
13	20/20	13	−15.63	10.7	-	-	+
14	20/20	20	−3.23	17.5	-	-	+
15	20/25	22.5	−25.73	12.8	-	n/a	-
16	20/30	25	−17.51	0	+	n/a	-
17	20/25	17	−4.30	0	+	-	-
18	20/20	23	−5.66	30.1	-	-	-
19	20/20	14	−1.61	25.6	-	n/a	+
20	20/25	15	−5.92	34.2	-	-	-
21	20/20	11	−1.45	28.3	-	n/a	-
22	20/25	13.6	−16.53	37.1	+	+	-

BCVA - best corrected visual acuity, uIOP - untreated intraocular pressure, MD - mean deviation, F/U – follow-up, APON - acquired pit of optic nerve, LC - lamina cribrosa.

^*^F/U duration indicates the interval between the initial and final SD-OCT.

OAG patients with retinoschisis had a higher IOP at SD-OCT scanning (IOP at detection of retinoschisis for eyes with retinoschisis) (P = 0.006) and worse visual field mean deviation (P = 0.010) than patients without retinoschisis. IOP fluctuation during the follow-up period (standard deviation of IOPs obtained during the follow-up) was higher in patients with retinoschisis with marginal significance (P = 0.056). There were no differences between OAG patients with retinoschisis and those without in terms of age, gender, untreated IOP, mean IOP during the follow-up period, axial length, central corneal thickness, and duration of follow-up. (Table 3)

**Table 3:** Comparison of clinical factors between glaucomatous eyes with retinoschisis and those without.

	Glaucoma with retinoschisis development (n = 22)	Glaucoma without retinoschisis development (n = 350)	P-value*
Age (years)	59.1±15.0	54.5±12.9	0.131
Gender (M/F)	11/11	174/176	0.979
Untreated IOP (mmHg)	18.4±5.6	16.7±5.1	0.154
IOP at SD-OCT scanning (mmHg)†	15.9±6.4	11.7±2.2	**0.006**
Mean follow-up IOP (mmHg)‡	13.1±3.3	12.5±2.0	0.360
IOP fluctuation (SD)§	2.7±2.7	1.6±0.8	0.056
Visual field MD (dB)	−9.3±8.3	−5.1±6.4	**0.010**
Axial length (mm)	24.8±1.6	24.3±1.3	0.202
Central corneal thickness (µm)	548.8±35.9	554.7±49.1	0.632
F/U duration (months)	20.0±14.1	20.2±5.6	0.927

IOP - intraocular pressure, MD - mean deviation, F/U – follow-up.

Values are shown in mean ± SD.

Statistically significant values are shown in bold.

* Comparisons were performed using independent samples t-test for continuous variables and chi-squire test for categorical variables.

† IOP at the time of retinoschisis detection for eyes with retinoschisis development.

‡ Average of the IOPs obtained during the observation period.

§ Standard deviation of the IOPs obtained during the observation period.

Univariate logistic regression analysis revealed a significant influence of higher IOP at SD-OCT scanning (Odds ratio  = 1.444, P<0.001), larger IOP fluctuation (Odds ratio  = 1.758, P = 0.003) and worse visual field mean deviation (Odds ratio  = 0.931, P = 0.014) on the presence of retinoschisis. In the multivariate analysis, only IOP at SD-OCT scanning was statistically significant (Odds ratio  = 1.418, P = 0.001). (Table 4)

**Table 4:** Factors associated with peripapillary retinoschisis in glaucoma group.

	Univariate			Multivariate		
	Odds ratio	95% CI	P value	Odds ratio	95% CI	P value
Age, per 1 year older	1.029	0.991–1.068	0.133			
Female gender	0.987	0.404–2.410	0.978			
Untreated IOP, per 1 mmHg higher	1.055	0.979–1.138	0.160			
IOP at SD-OCT scanning, per 1 mmHg higher*	1.444	1.200–1.738	**<0.001**	1.418	1.147–1.751	**0.001**
Mean follow-up IOP, per 1 mmHg higher†	1.131	0.941–1.360	0.188			
IOP fluctuation, per 1 mmHg greater‡	1.758	1.214–2.548	**0.003**	1.059	0.574–1.951	0.855
Visual field MD, per 1dB greater	0.931	0.880–0.986	**0.014**	0.941	0.876–1.011	0.097
Axial length, per 1 mm longer	1.326	0.858–2.048	0.204			
Central corneal thickness, per 1 µm thicker	0.998	0.988–1.007	0.631			
F/U duration, per 1 month longer	0.995	0.934–1.058	0.863			

CI – confidence interval, IOP - intraocular pressure, MD - mean deviation, F/U – follow-up.

Statistically significant values are shown in bold.

* IOP at the time of retinoschisis detection for eyes with retinoschisis development.

† Average of the IOPs obtained during the observation period.

‡ Standard deviation of the IOPs obtained during the observation period.

### Location, involved layers, and circular extent of the retinoschisis

**Figure 2:**
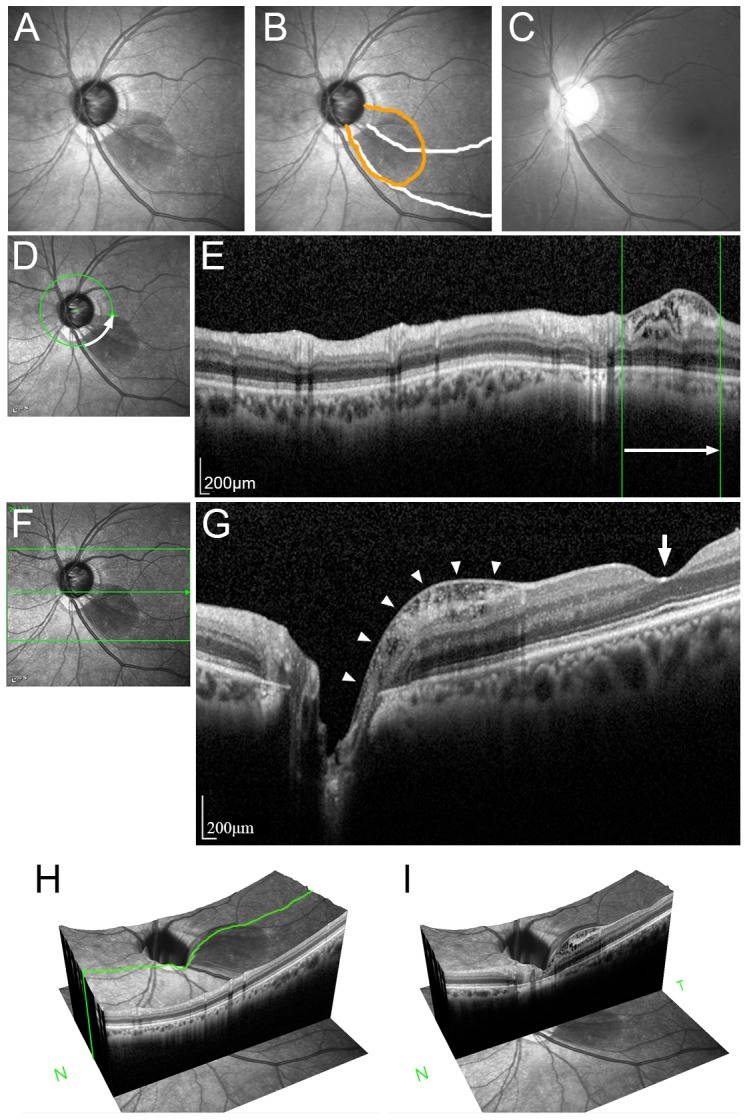
Infrared (IR) (A, B, D) and red-free fundus photograph (C), and SD-OCT (E, F, G, H) images in eyes with peripapillary retinoschisis. (A) In the IR image, peripapillary retinoschisis was identified as a dark, localized area with a smooth margin, which had a narrower base attached to the optic disc border, fanning out along the path of nerve fiber bundles. (B) Same image as (A) with labels. The area of the retinoschisis (orange line) overlaps with the retinal nerve fiber layer (RNFL) defect (white lines). (C) In the red-free fundus photograph, only the localized RNFL defect is seen. (D, E) The region of retinoschisis demarcated based on the circumpapillary B-scan image corresponding to the dark area shown in IR images. (F, G) Horizontal linear macular scan image. Note that the retinoschisis is localized in the peripapillary area (arrowheads), not involving the macular region (arrow). (H, I) Volume-rendered images. Note the elevation of the dark area at the locations of the retinoschisis.

In IR images, peripapillary retinoschisis was identified as a dark, localized area with a smooth margin. The dark area had a narrow base attached to the optic disc border, fanning out along the path of nerve fiber bundles (Figure 2A, B). The border of the retinoschisis was not distinct in red-free fundus photographs (Figure 2C). Retinoschisis was readily observed in the cross-sectional cpRNFL B-scan images at the location corresponding to the dark area shown in IR images (Figure 2D, Video S1). There was no macula involvement in any of the patients.

**Figure 3:**
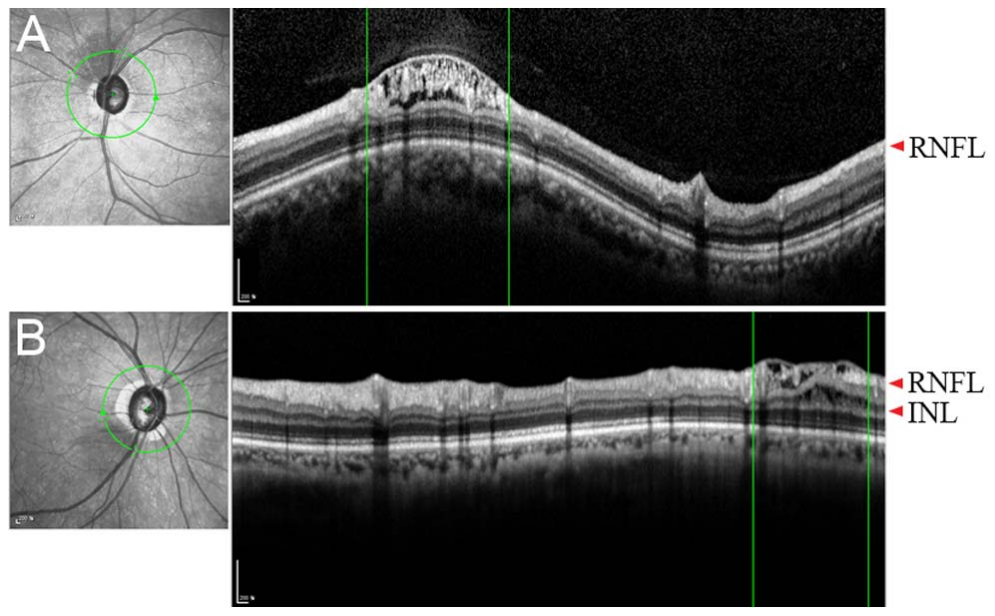
Circumpapillary B-scan images in cases 4 (A) and 11 (B). (A) Only the retinal nerve fiber layer (RNFL) is involved. (B) The RNFL and the inner nuclear layer (INL) are involved.

**Figure 4:**
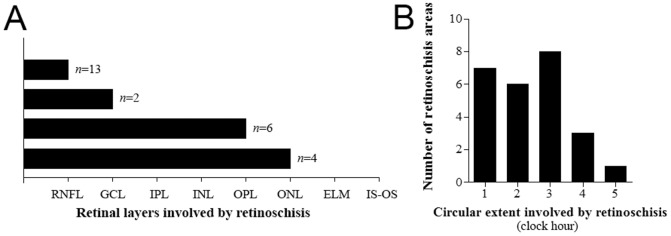
Retinal layers (A) and circular extent (B) involved in retinoschisis. (A) Involvement of only the RNFL was most frequent (13 retinoschisis). The RNFL was involved together with other layers in 12 retinoschisis. RNFL = retinal nerve fiber layer, GCL = ganglion cell layer, IPL = inner plexiform layer, INL = inner nuclear layer, OPL = outer plexiform layer, ONL = outer nuclear layer, ELM = external limiting membrane, IS-OS = junction between the photoreceptor inner and outer segments.

The involved retinal layers varied among retinoschisis. In 13 retinoschisis, only the RNFL was involved, while in 12 retinoschisis, the RNFL was involved together with other retinal layers. None of the layers outer than the ONL were involved in any of the eyes (Figures 3 and 4A).

The circular extent of the retinoschisis was 2.4±1.2 clock hours (mean ± standard deviation; range, 1–5 clock hours; Figure 4B). The location of the schisis overlapped with that of the localized RNFL defects (*n* = 17) or diffuse RNFL atrophy (*n* = 5) seen in the red-free photography in all eyes (Figure 2A–C).

### Association with acquired optic disc pits

**Figure 5:**
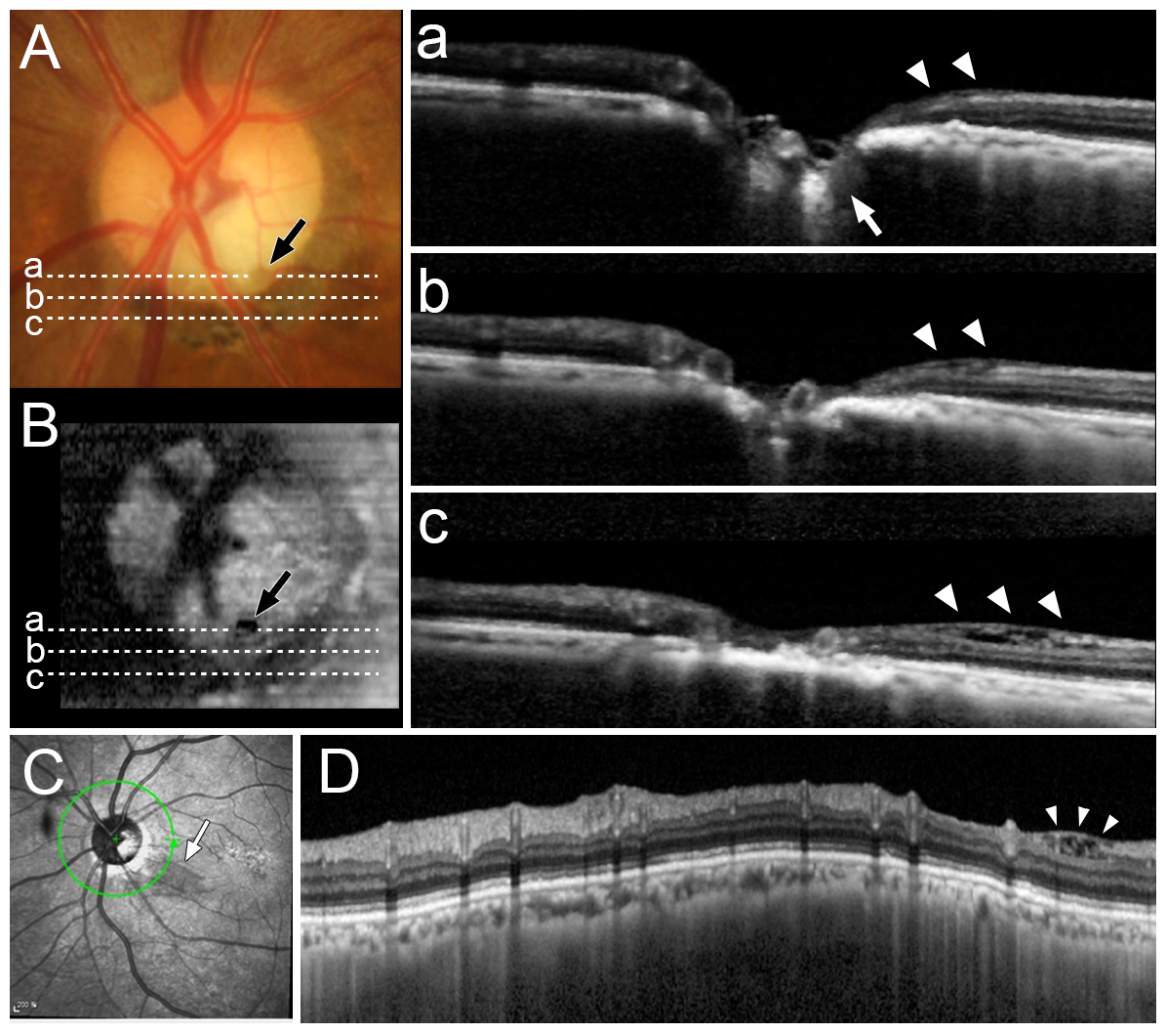
Optic disc photograph (A), en-face image (B), B-scan SD-OCT images (a, b, c), and infrared (IR) image (C) with circumpapillary B-scan image (D) in case 22. (A, B) A pit is seen near the disc margin (black arrows). (a, b, c) Horizontal B-scan images obtained at the location of pit (marked with dotted lines in A and B). Note the LC defect (large white arrows) at the location of optic disc pit. Retinoschisis is observed in all three B-scans (large arrow heads). (C, D) Retinoschisis is seen adjacent to the optic disc pit in both the IR (small white arrow) and the circumpapillary B-scan image (small arrow heads).

Acquired optic disc pits were observed in eight eyes on the stereo disc photography, all of which were located adjacent to the area of retinoschisis. Of these, EDI SD-OCT optic disc scans were available for seven patients. Optic pits were observed in EDI B-scan images and/or en-face images of five patients. The location of the pit was correlated with that of the retinoschisis (Figure 5).

### Clinical course of the retinoschisis

Of the 22 eyes, retinoschisis was observed at the first cpRNFL SD-OCT in 16 patients. In the remaining 6 patients, the retinoschisis was not observed initially but developed during the study period. Table 5 summarizes the IOP profile of these six patients around the development of their retinoschisis. The IOP did not differ between before and after development retinoschisis (*P* = 0.357, Wilcoxon signed-rank test).

**Table 5:** Intraocular pressure profile around the new development of retinoschisis (*n* = 6).

	IOP prior to retinoschisis detection (mmHg)	IOP at the time of retinoschisis detection (mmHg)
Case 1	11	10
Case 5	15	10
Case 7	12	10
Case 19	14	14
Case 20	12	14
Case 21	9	9

IOP - intraocular pressure.

**Figure 6:**
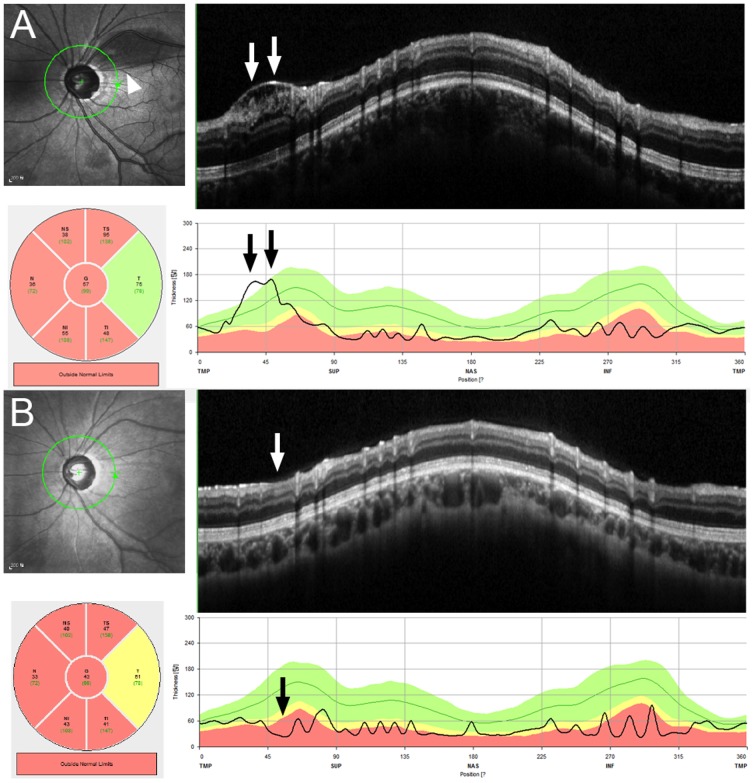
SD-OCT findings in a case of resolution of the retinoschisis (case 19). (A) The retinoschisis is observed in the B-scan image (white double arrows). (B) The retinoschisis is absent (single white arrow) in the image obtained 3 months later. According to the sector thickness map, superotemporal RNFL thickness was decreased from 95 (at the time of retinoschisis) to 47 µm (after resolution). The remarkable decrease in the retinal nerve fiber layer thickness in the superotemporal area is also seen in the TSNIT graphs (black arrows).

During the follow-up, resolution of the schisis was observed in nine eyes. In these 9 eyes, the SD-OCT RNFL thickness values at the schisis area notably decreased after the resolution when compared with the measurement at the time of retinoschisis (Figure 6). The resolution of retinoschisis was associated with additional IOP-lowering treatment, either by trabeculectomy (*n* = 2) or initiation of or additional IOP-lowering medication (*n* = 5; Table 6). The IOP after resolution of the retinoschisis was significantly lower than that at the time of its detection (*P* = 0.007, Wilcoxon signed-rank test).

**Table 6:** Intraocular pressure profile and anti-glaucoma intervention around the resolution of retinoschisis (*n* = 9).

	IOP-detection	IOP-resolution	Time to resolution (m)*	Intervention before the resolution
Case 2	16	13	15.2	Initiation of medication
Case 3	15	12	8.1	Additional medication
Case 8	15	11	5.1	Initiation of medication
Case 9	16	13	16.9	Initiation of medication
Case 10	15	14	24.3	No additional intervention
Case 12	26	10	3.5	Trabeculectomy
Case 13	13	10	10.7	Initiation of medication
Case 14	12	8	3.0	Trabeculectomy
Case 19	14	12	5.5	No additional intervention

IOP - intraocular pressure.

*Interval between the first detection and resolution of the retinoschisis.

## Discussion

This study investigated 22 glaucoma patients in whom retinoschisis had been observed in the peripapillary area, and characterized the topography and involved layers of the retinoschisis as evaluated by IR fundus and SD-OCT imaging. To the authors' knowledge, this is the first systemic investigation of the characteristics of peripapillary retinoschisis associated with glaucoma in a relatively large sample.

In the present study, the retinoschisis was found significantly more often in the glaucoma group (5.9 vs 0.5%). This finding may suggest that glaucomatous eyes are more prone to develop retinoschisis. Although the study design cannot address the prevalence of the retinoschisis (see the study limitation below), it appears that retinoschisis is not a rare phenomenon in glaucoma patients. It is likely that this condition is often overlooked in clinical practice because patients are often asymptomatic and the retinoschisis often appears normal on funduscopy;[9], [14] it also appears to be invisible in red-free photography (present findings). Clinicians are increasingly likely to encounter retinoschisis in glaucoma patients due to the expanded use of SD-OCT and IR imaging. Retinoschisis within the inner retinal layers is easily detected in IR images because the fluid in the schisis cavity blocks the signals reflected from the retinal pigment epithelium.[15]

Previous anecdotal reports of retinoschisis in glaucoma patients often described macular involvement. However, none of the patients in the present study had macular involvement. This may be related with the exclusion criterion of IPGS (visual acuity of <20/40). In addition, exclusion of the eyes with refractive error <-12.0D may be also related with the absence of macular retinoschisis in our patients. Macular retinoschisis has been frequently demonstrated in eyes with myopia beyond this criterion,[16], [17] although some reported patients had less degree of myopia.[18], [19] Meanwhile, of the 22 patients with retinoschisis, 13 were followed up for more than 1 year. During the follow up, retinoschisis had not expanded to the macular area in any patients. Furthermore, the retinoschisis were resolved in 7 patients of the 13 patients. Therefore, we consider that macular involvement by the expansion of retinoschisis is not a common finding, when the retinoschisis developed in the peripapillary region in glaucomatous eyes.

According to IR and horizontal B-scan images, the retinoschisis was universally attached to the optic disc, from which it spanned out. It overlapped with the RNFL defect seen in the red-free photography. In addition, acquired optic disc pits were often found at the location corresponding to the retinoschisis in some eyes. These findings suggest that the pathogenic mechanism of the retinoschisis occurring in glaucoma patients might be related with glaucomatous optic nerve damage (i.e., optic disc pit). We speculate that the damage to the LC may provide a conduit that allows fluid to travel from either the vitreous cavity [20], [21] or the subarachnoid space.[21]–[24]. We do not have clear answer on the pathogenesis of retinoschisis in eyes without optic disc pit. However, the topographic correlation between the retinoschisis and glaucomatous RNFL defect suggests the possibility that the retinoschisis in those eyes are still related with glaucomatous optic nerve damage. It has been suggested that microscopic interconnections between the vitreous space, RNFL and optic cup developed in the process of thinning of the optic nerve tissues may provide a conduit of fluid entrance to the retina in eyes with glaucoma.[8], [9], [25]

Vitreous traction also has been suggested as a triggering factor for the development of retinoschisis.[7], [9], [20], [26], [27] Studies have suggested that vitreous traction may facilitate the entering of liquefied vitreous fluid into retina. However, most studies have failed to find vitreous traction or detachment in eyes with retinoschisis.[7], [25], [28]–[30] In the present study, we could not evaluate the association of vitreous traction or detachment with retinoschisis. This was because the vitreo-retinal interface cannot be fully assessed using SD-OCT.[31] On the other hand, the present study did not find any differences between glaucomatous eyes with retinoschisis and those without, in age, gender or axial length, which has been suggested as factors related with posterior vitreous detachment formation.[32]–[38] Based on this finding, we speculate that the vitreous traction is not likely the primary mechanism for the retinoschisis in our study subjects.

It is of interest that the retinoschisis primarily involved the RNFL without serous detachment. The retinoschisis associated with congenital pits involves differing retinal layers and is often complicated with serous detachment. It is believed that the fluid enters different layers through the congenital pit.[24] Although there is no clear explanation for the preferential involvement of the RNFL in our patients, it might have been due to only the RNFL lying within the optic nerve head above the LC, hence resulting in a conduit for fluid being connected to the RNFL.

IOP elevation[7], [9], [27] or fluctuation[7], [8] has been suggested as a cause of retinoschisis. In line with these, IOP at the time of OCT scan was significantly associated in both the univariate and multivariate analysis. This finding may suggest that the mechanical stress driven from IOP play a role in the development of retinoschisis. However, in our six cases where the retinoschisis developed during the follow-up, the IOP did not differ between before and after this development, which suggests that retinoschisis can develop in the absence of IOP elevation and can be associated with other factors. However, the possibility that the eyes suffered from sustained, cumulative IOP-induced stress (despite the absence of IOP elevation) or IOP fluctuation that was not detected at the clinic cannot be ruled out.

The resolution of retinoschisis was associated with IOP reduction, which is consistent with Zumbro et al's finding that resolution of retinoschisis was noted after filtering surgery for uncontrolled glaucoma.[9] However, the amount of IOP reduction was often of small degree (1–4 mmHg) in the present study. Thus, we are not for sure that the resolution of retinoschisis was related with the IOP reduction. Further study involving large number of samples is needed to investigate the relationship between IOP fluctuation and the development and resolution of the retinoschisis.

Previous studies have shown that laser photocoagulation[4] or pars planar vitrectomy and gas tamponade[5], [9] were required to treat macular retinoschisis. In the present study, the retinoschisis often resolved during the follow-up. Although a prospective study is needed to define the long-term course of this entity, it seems prudent to follow up without surgical intervention to treat retinoschisis in glaucoma patients with peripapillary retinoschisis.

In eyes with peripapillary retinoschisis, the RNFL thickness was found to decrease remarkably after the resolution of retinoschisis, when compared with the measurement at the time of retinoschisis, at which the RNFL thickness was measured falsely thick. If a clinician simply looks at the measurement data without noticing the retinoschisis, he or she would consider such decrease as a rapid structural glaucoma progression. Given that retinoschisis is not rare, this issue may be clinically relevant. We propose that clinicians should rule out the resolution of preexisting retinoschisis when a sudden, unexpected decrease of the RNFL thickness is noticed at the RNFL thickness map, and the determination of glaucoma progression should be made based on other measures, such as optic disc and/or visual field examinations.

It is of interest to see whether the development of retinoschisis is associated with glaucoma progression. To see this, comparison of the optic disc and visual field parameters before the development of retinoschisis and after the resolution of the retinoschisis is needed. In our study sample, only 1 case showed the development and resolution during the follow up. Thus, our study cannot address this matter.

This study was subject to several limitations. First, this study was based on IGPS, which did not include consecutive patients. Thus, our data cannot be used to address the prevalence of retinoschisis. Second, only peripapillary retinoschisis was demonstrated in this study. However, macular retinoschisis has also been reported in the literature, and its clinical course may be different from that observed in our patients. Indeed, surgical intervention is needed in the macular schisis that develops in some glaucomatous eyes.[9] Third, this study was based on a review of the images obtained from the IGPS, which was not specifically designed for investigating the clinical course of the retinoschisis. Thus, the patients had different follow-up periods. As such, the rate of resolution could not be determined in the present study, which makes further longitudinal follow-up necessary.

In conclusion, 22 cases of peripapillary retinoschisis observed in glaucomatous eyes are detailed herein. The retinoschisis overlapped with the RNFL defect and was associated with acquired optic disc pits in some eyes. The condition often resolved spontaneously while the patient was receiving IOP-lowering treatment, without causing serious visual loss. Care should be taken not to overestimate the RNFL thickness in eyes with retinoschisis, and also not to misinterpret the resolution of retinoschisis as a rapid structural glaucoma progression.

## Supporting Information

Video S1 **3-dimensional volume-rendered image of the eye shown in** **Figure 2** **.** Note the elevation of the dark area at the location of retinoschisis.
(AVI)
